# Physical Activity Participation among Children and Youth with Mental Health Symptoms: Clinician Perspectives

**DOI:** 10.3390/children11070880

**Published:** 2024-07-20

**Authors:** Madeline Crichton, Julie Vu, Barbara Fenesi

**Affiliations:** Faculty of Education, Western University, London, ON N6G 1G7, Canada; mcricht3@uwo.ca (M.C.); jvu32@uwo.ca (J.V.)

**Keywords:** physical activity, mental health, clinician perspectives, barriers to physical activity

## Abstract

Background/Objectives: Physical activity supports mental health and well-being in children and youth. However, there are significant barriers to physical activity participation among individuals impacted by mental health disorders. This study investigates these barriers through the perspective of mental health clinicians who support children and youth. Methods: Fourteen mental health clinicians, including registered professional psychologists, psychotherapists, and social workers, were interviewed in a semi-structured format. Qualitative content analysis was performed to identify key themes, including both barriers and facilitators to physical activity. Results: Content analysis revealed that clinicians perceive both internal and external barriers and facilitators to their clients’ participation in physical activity. Barriers included intrapersonal factors, such as the presence of depression, anxiety, or eating disorder symptoms; lack of motivation; and negative self-talk, as well as factors related to the influence of caregivers, financial limitations, screen time use, environmental and cultural factors, and lack of time. Facilitators included enjoyment of physical activity, knowledge about the benefits of physical activity, and caregiver participation. Conclusions: Mental health clinicians demonstrated clear knowledge about the barriers to and facilitators of their clients’ participation in physical activity. These findings provide valuable insights that can be used to support children and youth experiencing mental health difficulty to access the beneficial effects of physical activity.

## 1. Introduction

The impact of physical activity on mental health has become increasingly well known in recent years [1,2,3,4,5]. Recent research has gone so far as to suggest that physical activity can be as impactful as antidepressant medication in the treatment of mood disorders [6,7]. Given the positive effects of physical activity on mental health, it holds potential as a low-cost and low-risk option for promoting mental health [8]. This may be especially valuable for children and youth given that mental health needs are rising among this population, with the current services inadequately available and affordable to meet demand [9,10]. Around 20% of youth experience symptoms of mental disorders, a number that has likely gone up since the COVID-19 pandemic [11,12]. In Canada, emergency department visits for mental health concerns among young people aged 5 to 24 have risen by 75% since 2006 [13], yet over 40% of Canadians with mental health concerns report that their needs have not been met [9]. At the same time, less than 7% of children meet the World Health Organization (WHO) recommended standard of 60 min of moderate-to-vigorous physical activity per day [14,15,16]. Most Canadian children and teens spend more than 50% of their waking hours sedentary [17], and the number of steps children take per day has trended downward over time [18]. Given the low rates of physical activity and the high rates of mental health symptoms among youth, it is important to understand the barriers to and facilitators of physical activity among youth who experience mental health difficulties.

The impact of physical activity on mental health appears across symptom types and in diverse populations [19,20,21]. Physical activity has been linked to improved affect, mood, emotional regulation, self-efficacy, motivation, and overall well-being, as well as decreased depressive and anxiety symptoms [1,8,22]. Children and adolescents who engage in more physical activity are less likely to experience mood disorders, including major depression and bipolar disorder [23], and to report higher rates of life satisfaction than their less-active peers [24]. Physical activity also supports improved executive functioning, attentional control, and self-regulation, especially in children and adolescents with ADHD [25,26]. There are several mechanisms that appear to contribute to the link between physical activity and mental health, on both biological and psychosocial levels of interaction. In terms of biological processes, physical activity upregulates the brain-derived neurotropic factor and the release of endorphins in the brain [27,28]. These processes are thought to lead to positive structural and functional changes in the brain that support learning, memory, and emotional regulation [29]. On the psychosocial level, youth participation in physical activity increases social connection, which may, in turn, lead to improvements in mental health [30], as well as a reduction in specific anxiety symptoms through exposure to new experiences [31].

However, participation in physical activity for individuals experiencing mental health challenges is often easier said than done. The nature of mental health symptoms themselves can create a barrier to physical activity participation [32,33]. For example, the symptoms of depression include low motivation or interest in activities, fatigue or lack of energy, and low mood, all of which can be linked to a lower likelihood of physical activity participation [34]. Similarly, symptoms of anxiety, particularly social anxiety, may lead to a reluctance to participate in physical activity in locations where it is possible to be observed by others, and the executive dysfunction symptoms (i.e., forgetfulness and distractibility) that characterize ADHD may impair one’s ability to regularly participate in physical activity [35,36]. Furthermore, there are additional barriers to physical activity participation among children with mental health disorders, such as low self-efficacy, low motivation, lack of parental or social support, lack of training for school professionals, and a lack of inclusive physical activity programming [37]. This then creates a feedback loop, wherein individuals faced with mental health symptoms become less likely to be physically active, which can, in turn, lead to worsening mental health [32,38].

Physical activity promotion efforts have been ongoing for several decades [39]. During this time, it has become clear that achieving behaviour change with respect to physical activity is a complex and multi-faceted process [40]. Several theoretical frameworks have been proposed to describe the range of internal and external influences on behaviour required to support engagement in physical activity [41]. Two such frameworks are the dual process theory and the socioecological model. The dual process theory posits that there are two main behavioural determinants—reflective and automatic processes—and that individuals tend to act in line with automatic processes, unless sufficient reflective motivation exists to override them [41,42]. In the context of physical activity, the dual process framework would submit that automatic processes (e.g., habits and non-conscious self-schema) may lead to sedentary behaviour when that is the individual’s existing routine or if there is a self-schema that they are not a person who exercises, for example. Under this theory, the individual would remain sedentary until and unless they engaged in a deliberate process of reflecting on the knowledge and values supporting physical activity participation, which would then lead to motivation. Once habits of physical activity participation are developed, it can then become a more automatic process. The socioecological model posits that behaviour is a function of individual and environmental influences [41,43]. In the context of physical activity, socioecological work has primarily focused on the environmental level, given that other existing frameworks tend to focus on individual and social factors. Physical activity levels have been linked to environmental features, such as mixed-use areas, pedestrian network connectivity, and quality of physical activity infrastructure [44,45]. Environmental features may have the largest effects on those who are less active, and intentions and behaviours are more likely to be aligned when recreational facilities are nearby [46]. Taken together, these frameworks help explain the multiple levels of influence that can be exerted on physical activity behaviour. For those with mental health difficulties, the individual and automatic influences may appear most obvious. However, the impact of social and environmental influences, as well as the potential role of clinicians in facilitating reflective processing, provide promising avenues to address barriers and facilitate access to the full benefits of physical activity.

Indeed, support and recommendation from a professional is associated with increased participation in physical activity [47,48]. Prior work has identified that mental health clinicians are aware of the benefits of physical activity and that approximately two-thirds of clinicians routinely discuss physical activity with clients [49,50,51]. They are most likely to recommend physical activity to clients presenting with symptoms of depression, anxiety, and stress [52,53]. Clinicians may find a role within the dual-process model by facilitating reflective processes and supporting the adoption of new routines. They may also provide support across levels of the socioecological model by enhancing individual motivation and troubleshooting environmental barriers. Clinicians’ attitudes towards physical activity as part of mental health care have increased in positivity over time [50,51,54], and clinicians report interest in further training and in opportunities to increase the role of physical activity in their clinical practice [49,53].

However, the integration of physical activity into mental health care is not universal. Many clinicians are not discussing physical activity or only doing so occasionally [50,55]. Clinicians identify barriers related to their own confidence, training, and knowledge as impeding the discussion of physical activity within their practice [49,52]. They also identify barriers within their clients, such as a belief that clients will not be motivated to participate in physical activity and that they may lack the resources to engage in physical activity, as well as concerns about certain presenting issues, such as trauma and eating disorders [51,56,57]. It is therefore important to understand and consider clinician-identified barriers to their client’s physical activity participation, as this may impact whether and how often clinicians discuss physical activity with particular clients. At the same time, clinicians do likely have good insight into the barriers faced by their clients [58] and are likely to be reliable reporting sources [59]. External reports of behaviour are generally considered valid sources of information [60,61], especially when children are concerned, as young children may lack insight or, at the very least, the ability to effectively communicate their insight. Clinicians may also have additional insight into the barriers and facilitators faced by their clients due to their training in psychology [61]. As such, there is tremendous value in clinician perspectives of the barriers to and facilitators of engaging in physical activity faced by their clients. Understanding these barriers and facilitators may provide insight into strategies that clinicians can use to assist their clients in participating in physical activity so that they can reap the positive mental health benefits.

Therefore, the current study aims to investigate clinician-identified barriers to and facilitators of physical activity participation among children and youth involved with mental health services. The purpose of this study is to better understand the factors impacting physical activity participation among youth with mental health needs, as well as to gain insight into strategies that may be beneficial for clinicians promoting physical activity among their clients. It is hypothesized that clinicians will be able to describe multiple barriers and facilitators, and these may include internal barriers and facilitators (such as mood, anxiety, or motivation), as well as external barriers and facilitators (such as time, access to resources, or environmental constraints). The clinician-identified barriers and facilitators are expected to be broadly consistent with client-identified barriers and facilitators that have been previously described in the literature.

## 2. Materials and Methods

### 2.1. Participants

Mental health care providers in the province of Ontario were invited to participate in an interview study. Eligibility requirements included that clinicians had to be registered with a professional college in Ontario and had to do at least some of their clinical work with children or adolescents. Participants were recruited via advertising on lab social media pages and in professional Facebook groups and email listservs.

A total of fourteen clinicians participated in the study. Participants included seven registered psychotherapists, five registered psychologists, and two registered social workers. There were two male and twelve female participants. Eleven clinicians worked exclusively in private practice, one worked exclusively in a hospital setting, and two worked in both private practice and hospital settings.

### 2.2. Procedure

Prior to engaging in the interviews, participants were contacted by email and asked to confirm their professional registration and their work with children to ensure eligibility. Participants provided informed and voluntary consent. Each participant engaged individually in a semi-structured interview with a researcher (MC) via Zoom video conferencing. The interviews lasted approximately 30 min (range = 20 to 38 min), and the audio was recorded for transcription using the platform’s audio recording service. Participants were compensated for their time in the form of a 10 CAD gift card. This may have incentivised participation; however, the amount of compensation was determined to be a token amount that would minimize the risk of biasing results. The study received approval from the institution’s research ethics board.

### 2.3. Materials

The interviews were conducted using a semi-structured format. Interview questions were prepared by the research team with the aim of eliciting responses that would answer the research questions as described above. The interviews consisted of 4 primary questions that were asked verbatim by the researcher (see Appendix A). These questions were intentionally broad, so as not to limit participant responses. Additional follow-up questions were asked flexibly based on participant responses and when necessary to gain more detailed information.

### 2.4. Qualitative Data Analysis

The data collection and analysis were centered within a post-positivist paradigm, which acknowledges that researchers will necessarily hold background knowledge and biases that may impact their observations and interpretations of data [62]. Given that both the primary researcher and the research assistant involved in coding were graduate trainees in clinical mental health programs whose training experiences, clinical frameworks, and background knowledge could impact their perceptions of the data, the post-positivist paradigm was believed to be most appropriate.

Inductive content analysis was used to analyze the interview responses. Content analysis describes a procedure for compressing text into categories based on specific content codes [63,64]. Since content categories were derived from the data, the analysis was inductive. The content analysis procedure was used to identify and report recurring themes in the interview data to understand trends and commonalities across participants.

Interviews were initially transcribed using Zoom’s built-in transcription service. The transcripts were then manually checked against the audio recordings. Two researchers read the transcripts to become familiar with the data. Following the familiarization phase, the same two researchers collaboratively developed a preliminary codebook to categorize the data based on emerging themes. The researchers consulted on the resulting codebook and discussed the success of the codebook in capturing key themes, and a final codebook was developed. The codebook was then reapplied to all transcripts using MAXQDA (V24), a qualitative analysis software program. Frequencies were generated for each theme. Each researcher independently reviewed each transcript, and any discrepancies were resolved through discussion until consensus was achieved.

## 3. Results

The data are presented separately for barriers and facilitators, with each section divided by themes and subthemes identified in the data. The themes and subthemes are presented in order of frequency, determined by the number of times participants discussed each theme in the interviews. Illustrative quotations are provided from the interview transcripts. Table 1 provides a frequency summary of the barriers and facilitators to physical activity participation that were identified by clinicians.

### 3.1. Clinician Identified Barriers to Physical Activity Participation

#### 3.1.1. Theme 1: Intrapersonal Factors (Frequency 64)

Clinicians identified intrapersonal client-related barriers to physical activity participation. This included the discussion of clients’ mental health symptoms or other intrapersonal factors that negatively impacted their ability to be active. These factors (subthemes) included depression and anxiety, lack of motivation, negative self-talk and cognitive rigidity, and eating disorders.

Subtheme 1a: Depression and Anxiety (Frequency 19).

Clinicians expressed that their clients’ depression and anxiety symptoms were themselves barriers to participation in physical activity. For example, one clinician described a client who “was into Taekwondo and got really depressed, and also had social anxiety, and it just melded together. And then they couldn’t do their Taekwondo anymore.” (Participant 7). As another clinician described a client:

“He used to love to go to the gym and do things that were physically active. But he’s been having a really hard time lately because he’s been feeling quite depressed... it’s a vicious cycle of physical activity could really benefit him by boosting endorphins, all that sort of stuff. But since he has a lack of motivation to get there in the first place, he tends to stay in that depressed state instead (Participant 10)”.

Subtheme 1b: Lack of Motivation (Frequency 15).

Several clinicians also reported that their clients lack motivation to be active, which was often expressed as related to mental health symptoms or to a lack of familiarity with physical activity. Clinicians noted “I do feel the challenge really is getting people to commit and start.” (Participant 1); “The motivational piece is where I think they get stuck.” (Participant 14); “If it’s new for them and they don’t have social support around it, I find there’s very little motivation.” (Participant 8).

Subtheme 1c: Negative Self-Talk and Cognitive Rigidity (Frequency 11).

Clinicians also described clients talking negatively about themselves or their ability to participate in physical activity and having rigid perspectives about being able to engage in physical activity. As one clinician put it, “That mindset of like, I don’t exercise, or that’s not for me, it’s tough to overcome.” (Participant 1). Another clinician described the difficulty of introducing the idea of physical activity to clients with an inflexible mindset: “There’s often a fair bit of rigidity and cognitive rigidity. And some of these kids see themselves as I’m good at X, and that’s all I want to do. Often it’s being on screens, or gaming.” (Participant 2).

Subtheme 1d: Eating Disorders (Frequency 5).

Several clinicians described the unique barriers to physical activity in clients with eating disorders. One clinician said “With eating disorder recovery, a lot of times physical exercise, intensive exercise, needs to be stopped for a period of time as it is not safe for them to be going to the gym or playing sports or even running or things like that.” (Participant 5).

#### 3.1.2. Theme 2: Influence of Caregivers (Frequency 29)

Clinicians identified barriers to physical activity participation related to the influence of caregivers. Clinicians noted that, with child and adolescent clients, parents or caregivers can play a significant role in access to physical activity. The subthemes included caregiver attitude to physical activity and clients’ dependence on caregivers.

Subtheme 2a: Caregiver Attitude to Physical Activity (Frequency 19).

Clinicians discussed how the role of caregivers’ attitudes and beliefs about physical activity can influence clients’ physical activity. For example, clinicians said “I think more often it’s habit and the culture in the family. If parents are pretty sedentary or they’re staring at screens all day, it’s sometimes really hard to engage in considering an alternative to that.” (Participant 2); “I’d say maybe parental attitudes is how I might flag that. If parents aren’t seeing [physical activity] as one of the things that they thought their kid was coming to therapy for, it might be harder to organize that and get that to happen.” (Participant 3).

Subtheme 2b: Dependence on Caregivers (Frequency 8).

Clinicians also discussed the practical reality of caregivers as the gatekeepers to access to physical activity opportunities for their children. Regarding young children, one clinician said “Young kids don’t have control over their schedule or selections, right? So, they’re not the ones going onto the city website and signing up for swim lessons or they’re not the one that’s going to the local soccer club and signing up.” (Participant 12). Another clinician considered adolescents: “For example some clients... they’re in high school or something like that and they want to go to the gym, they can’t because maybe they don’t drive.” (Participant 11).

#### 3.1.3. Theme 3: Financial Limitations (Frequency 16)

Clinicians also frequently discussed financial barriers that may arise for clients and their families. Some clinicians reported “So I would say that the biggest barrier has been financial for some kids to be able to get into activities or to do a certain sport.” (Participant 3); “At the hospital I saw people of a more diverse kind of economic situation, where it’s not affordable to necessarily play hockey or any of these sports that require a lot of expense.” (Participant 1).

#### 3.1.4. Theme 4: Screen Time (Frequency 12)

Several clinicians also described the role of screens and excess time on screens that can make engaging in physical activity more challenging to their clients. One clinician described that clients will avoid engaging in physical activity because “they’ll say they’re tired. I think they stay up gaming, or whatever really late. And so there’s not a great routine.” (Participant 8). As another participant put it:

“I meet a lot of adolescents who are social media addicted. And some of them stay at home scrolling for hours and hours... So I think the challenge is to break that habit. I do really suggest going outside for a bit instead of screentime. But sometimes they find it difficult to break that habit, because they’re so comfortable with it. And that’s their main coping mechanism.” (Participant 13)

#### 3.1.5. Theme 5: Environmental and Cultural Factors (Frequency 11)

Several clinicians discussed the role of their clients’ physical and cultural environments as potential barriers to accessing physical activity opportunities. One clinician described the role of her client’s physical environment as follows: “She lives in a rural part of the province. And so there really is nowhere for her to walk...she has to be really intentional as opposed to someone who lives in a neighborhood where there are sidewalks.” (Participant 6). Another clinician discussed the role of being a newcomer to Canada: “I definitely think for newcomers to Canada, there are a lot of barriers to accessing physical activity for their kids. And there are so many [activities] that are reasonably priced, but you really do need to know how to use the system.” (Participant 9).

#### 3.1.6. Theme 6: Lack of Time (Frequency 9)

Clinicians also discussed the role of time in the schedules of busy children and families to engage in physical activity. One clinician said “For kids, especially families with multiple children, it’s harder for them to dedicate time to exercise or [physical] activities.” (Participant 4). Another reported “Sometimes I think about single parents, and how it may be a lot harder for them time wise.” (Participant 9).

### 3.2. Clinician Identified Facilitators to Physical Activity Participation

Clinicians discussed several possible facilitators of physical activity engagement among their clients. These included enjoyment of physical activity, knowledge of physical activity benefits, and caregiver participation in physical activity.

#### 3.2.1. Theme 7: Enjoyment of Physical Activity (Frequency 22)

Clinicians noted that their clients who have an existing enjoyment or interest in physical activity, and those who were able to identify physical activity that they enjoyed, were more likely to participate more frequently in physical activity to support mental health. As one participant said, “If they really hate an activity, then they get the chance to be like, oh, but I like this activity. And then you get to let them explore those things, which makes physical activity just so much more exciting.” (Participant 5). Another participant described the way they discuss physical activity when introducing it to their clients: “Can we make it pro social? Can we make it fun instead of going to the gym, or counting the number of sit-ups you’re doing? Can you join a yoga class with a friend? Can you do that type of thing, try to find more team-based or group-based activities?” (Participant 8).

#### 3.2.2. Theme 8: Knowledge of Physical Activity Benefits (Frequency 8)

Clinicians also discussed the importance of clients’ knowledge about how physical activity may be beneficial to their mental health and well-being. One clinician described the power of this knowledge for their adolescent clients:

“Usually once they are able to make that link or we’re able to talk about the link between physical activity and mental health, that will tend to encourage them once they really pick up on that. Especially teenagers when they’re like, oh wait, that actually did help. As much as it’s maybe not super fun to go to the gym, that can really help.” (Participant 10).

Another clinician discussed how this knowledge can be impactful throughout life. “If we can equip our children and youth with this type of information and help them practice it, it will help them in their adult years because there are so many adults who don’t understand how this works.” (Participant 6).

#### 3.2.3. Theme 9: Caregiver Participation in Physical Activity (Frequency 6)

Several clinicians noted that, in their experience, when caregivers participate in physical activity, this can facilitate physical activity in their children. As one participant said, “I think within the culture of families, if parents are modeling and showing their kids by example that being physically active is really beneficial for health, for mental health, for family engagement. Like, there’s so many ways it could be used.” (Participant 2).

## 4. Discussion

This study aimed to understand the barriers to and facilitators of physical activity participation for children and youth accessing mental health care through the perspective of clinicians. As hypothesized, clinicians identified several barriers, including both internal client-related barriers and external barriers related to factors such as the clients’ caregivers and environments. Facilitators related to enjoyment, knowledge, and caregivers were also identified. The following section will discuss the identified themes and will consider how these results can inform future clinical practice.

### 4.1. Clinician Identified Barriers to Physical Activity Participation

#### 4.1.1. Intrapersonal Factors

Clinicians identified several intrapersonal or within-client factors that contribute to difficulty engaging in physical activity participation. This is consistent with previous works showing that mental health symptoms themselves create a barrier to physical activity [32,33]. Clinicians described these factors in several ways, including anxiety and depression symptoms, lack of motivation, negative self-talk and cognitive rigidity, and eating disorder symptomology. Clinicians described client experiences such as feeling stuck and unmotivated due to depression, social anxiety impacting the ability to engage in physical activity in group settings, eating disorder symptoms being triggered by exercise, and inflexible thinking patterns leading to difficulty trying new things. Many of these descriptions are consistent with the feedback loop described in the previous literature, in which mental health symptoms make physical activity more difficult, leading to a lack of physical activity, which, in turn, worsens mental health symptoms [35,38].

The first subtheme, depression and anxiety, reflects clinicians’ recognition that these common symptoms impact physical activity participation. For individuals with depression, they may feel lethargic and lack energy to engage in physical activity; they may also feel hopeless that trying anything new will lead to improvements in their mood [34]. Indeed, clinicians noted that it can be challenging to support their depressed clients in being active. However, they also described seeing improvements in the moods of clients who were able to break the cycle and become active, consistent with recognized trends [1,23]. Clinicians also reported challenges engaging their anxious clients in physical activity. They reported that clients with social anxiety in particular tended to be reluctant to participate in physical activity in social or public settings due to fears about judgement. For many individuals, depression and anxiety cooccur, which may compound the difficulties described by clinicians [65].

Clinicians frequently discussed the second subtheme of motivation as a factor impacting their clients’ ability to engage in physical activity. Several clinicians made the point that getting started was the hardest part for their clients and that participation became easier once habits had formed. Clinicians also emphasized the importance of starting with short bouts of light activity, such as going for a brief walk. Engaging in physical activity was also seen as easier if it was looked at as a fun, social activity and was specific to their client’s interests or preferred activities. This is consistent with self-determination theory, which considers both intrinsic and extrinsic motivators within the context of social and cultural factors [66]. Clinicians appeared to intuit that individualized, enjoyable activities would be more intrinsically motivating to clients. The points that clinicians in this study made about the difficulty of finding motivation to engage in physical activity, and the strategies they identified to increase motivation, are largely consistent with the previous literature [67,68], suggesting that clinicians are knowledgeable about factors related to motivation to engage in physical activity.

Clinicians also described the role of negative self-talk and cognitive rigidity as barriers to physical activity engagement. They noted that many of their clients presented with biased perspectives about themselves and their physical activity habits. Cognitive rigidity describes a chronically inflexible mindset that has been identified as a maintenance factor in psychopathology [69]. Negative self-talk is a related concept that is a common feature of mood and anxiety disorders and refers to negative self-statements [70]. The identification of these cognitive factors by clinicians as barriers to physical activity participation again points to the reciprocal relationship between mental health symptoms and physical activity, with traits related to the specific pathology contributing to an individual’s difficulty participating in physical activity. Clinical work focused on flexible thinking and positive self-talk may pay dividends by increasing clients’ willingness to participate in physical activity, in addition to the already established benefits of cognitive flexibility in symptom reduction [71].

Clinicians also regularly brought up the unique barriers faced by their clients with eating disorder symptomatology. Problematic exercise is a common concern in eating disorder treatment and recovery [72]. Guidelines suggest a careful reintroduction of physical activity for underweight patients that emphasizes the social and pleasurable aspects of physical activity and discourages solitary activity [73]. Several clinicians in this study reported that they regularly serve populations with eating disorder symptoms and would not approach discussions of physical activity in the same way they would with their other clients. A more cautious approach would be necessary and would need to include addressing problematic beliefs related to physical activity and the promotion of enjoyable, social activities.

#### 4.1.2. Influence of Caregivers

Clinicians also identified barriers to their clients’ participation in physical activity related to the influence of caregivers. Given that this study focused on children and youth, clinicians frequently acknowledged the role that parents or other caregivers play in their children’s access to physical activity. The primary themes were related to caregiver attitudes and dependence on caregivers. Clinicians perceived that caregivers with more positive attitudes towards physical activity would be more likely to promote physical activity in their children. This belief aligns with the evidence—parent and child physical activity are indeed correlated [74]. Caregiver perceptions related to therapy and expectations for treatment were also identified by clinicians as a potential barrier. It may be that caregivers who bring their child for talk therapy are skeptical of the benefits of physical activity and do not see it as central to treatment. Again, clinicians identified that caregivers who see physical activity as less important would be less likely to promote it for their children, which may point to the potential impact of caregiver-directed psychoeducation about physical activity and mental health.

Clinicians also described the practical barriers associated with their clients’ dependence on caregivers. Children typically rely on their caregivers to enroll them in sports or other programs, to provide the funds and transportation to engage in these activities, or even for the organization of informal activities, such as family walks. Clinicians noted that, unlike for their adult clients, children would have less control over their engagement in physical activity. While a clinician working with an adult would be able to directly problem solve around physical activity with their client, those working with children or youth may require parental involvement in these discussions.

#### 4.1.3. Financial Limitations

Financial limitations were also identified by clinicians as a potential barrier to physical activity participation. Clinicians noted that sport participation can be costly and may be out of reach for some families. Although not uniform, there is support for the notion that adolescents of lower socioeconomic status (SES) are less physically active than their higher SES counterparts [75]. Disparities are particularly strong for sport participation but are less pronounced for general daily movement, such as active transport [76]. This has implications for clinical practice, in that the promotion of daily movement habits may be more likely to be accessible and sustained in lower SES clients than organized sport participation.

#### 4.1.4. Screen Time

Another barrier clinicians frequently identified was the role of screen time for their clients. Clinicians described that their child and youth clients often spend time engaging with technology at the expense of other activities. Excessive screen time is linked to lower rates of physical activity; lower physical strength; deficient sleep; and increases in depression, anxiety, and emotional reactivity [77,78,79]. The association between mental health symptoms and screen time appears to be bidirectional. Symptoms of depression, for example, may lead to excessive solitary screen time use [80]. This further complicates the challenge of reducing screen time and increasing physical activity. Clinicians in this study seemed to be cognizant of the negative impacts of screen time while also finding the reduction in screen time to be a challenging task for children and their families. However, it is possible that, by targeting physical activity as an intervention strategy, there would be a multi-faceted benefit, in that physical activity targets symptoms directly while also potentially reducing screen time and leading to a further reduction in symptoms.

#### 4.1.5. Environmental and Cultural Factors

Clinicians discussed a variety of barriers related to environmental and cultural factors. Factors related to physical environments included a lack of access to physical activity programs and facilities in rural areas, apartment living and lack out outdoor play spaces, and unsafe neighbourhoods for outdoor activities. The cultural factors mentioned included difficulty navigating systems for newcomers and religious constraints on physical activity, such as avoiding mixed-gender active spaces. It was clear that clinicians were considering numerous aspects of their clients’ personhood in identifying barriers and determining solutions. In general, clinicians expressed an interest in addressing the unique needs of each of their clients collaboratively and flexibly to promote physical activity. This aligns with the existing guidelines for family physicians, which endorse individualizing physical activity recommendations to each patient’s needs and circumstances [81]. Mental health clinicians seem well poised to follow suit and provide collaborative recommendations based on environmental and individual factors. However, specific cultural factors related to physical activity motivation require additional study. Some work has established cultural differences in reasons that individuals feel motivated to engage in sports or other activity [82]; however, these differences are not yet well established enough to provide specific guidance for professionals, and additional study is required.

#### 4.1.6. Lack of Time

Another barrier noted by clinicians was the lack of time to engage in physical activity. They described that large families balancing the needs of several children may have difficulty supporting the physical activity participation of a specific child. Single parents, shift workers, and others with more complex schedules may also have difficulty carving out time to support physical activity. Among youth themselves, balancing extracurriculars, homework, and social activities may limit leftover time to participate in physical activity. Among adults, time is a frequently cited barrier to physical activity participation [83], and it follows that this may extend to supporting their child’s physical activity. However, other work has found that both active and non-active individuals perceive similar barriers to physical activity participation, indicating that time-related barriers can be overcome when there is sufficient motivation and prioritization of physical activity [84]. Thus, although time may not be a workable barrier within the context of psychotherapy, clinicians may be able to support the prioritization of physical activity when appropriate within a therapeutic context.

### 4.2. Clinician Identified Facilitators of Physical Activity Participation

#### 4.2.1. Enjoyment of Physical Activity

In addition to the barriers discussed above, clinicians also identified several facilitators, including the enjoyment of physical activity. Some noted, for example, that, when individuals had a pre-existing interest in sports or other physical activities, it was easier to support them to reengage in physical activity, even if they had stopped during their mental health symptoms. Clinicians also described that it was easier for their clients to build habits when they were engaging in activities that they enjoyed and chose for themselves. This was often discussed in the context of clinicians working collaboratively with their clients to develop plans for physical activity that would be realistic and doable. Enjoyment of physical activity is correlated with higher physical activity participation, as well as greater self-efficacy [85,86]. This may contribute to individuals with poorer self-efficacy, which is associated with poorer mental health [87], also experiencing a lower enjoyment of physical activity and lower physical activity participation. Confidence and self-efficacy related to physical activity may be an appropriate therapeutic target when increased physical activity participation is a goal of therapy, along with a focus on enjoyable physical activity.

#### 4.2.2. Knowledge of Physical Activity Benefits

Another facilitator described by the clinicians was their clients’ knowledge about the benefits of physical activity. Clinicians reported that clients who were aware of and understood the beneficial effects of physical activity for mental health were more willing to engage in physical activity. Some clinicians also described experiences where their clients were able to learn through experience that physical activity was beneficial for them, and this led to a greater willingness to engage in physical activity. Others discussed the notion that the physical health benefits of activity are more widely known and are often discussed in schools, while the mental health benefits may be less well known. Finding opportunities for psychoeducation within the therapy context may be beneficial, as well as greater community outreach by the mental health field to increase awareness.

#### 4.2.3. Caregiver Participation in Physical Activity

Although the factors related to caregivers were also identified as barriers, clinicians reported that caregiver participation also has the potential to facilitate physical activity. Several clinicians described the role that parents or caregivers could play in supporting their child’s physical activity. Clinicians discussed that, when caregivers provide a model for physical activity participation, this may encourage physical activity among their children. They also described a belief that parents could facilitate physical activity by organizing family activities, such as going for a walk or running as a family. As noted previously, physical activity among parents and children is correlated [74], lending support to the perception of the clinicians in this study that caregivers have an influence over their child’s physical activity for better or for worse.

### 4.3. Limitations and Future Directions

This study contributes a new perspective on the barriers to and facilitators of physical activity in youth facing mental health difficulties—that of the clinicians who support them. The clinicians who participated in this study provided valuable and well-informed insights. However, this study is not without limitations. The sample was self-selected, and the clinicians who participated were likely those with the greatest interest in physical activity and its role in mental health care. Thus, the results of this study may overestimate the knowledge and interest of the typical clinician in terms of their approach to discussing physical activity as part of their practice. The sample was further limited in that the clinicians primarily worked in private practice, with some working in hospital settings. Other work settings (e.g., schools, community agencies, and the justice system) were not represented, meaning that the results may not account for all barriers faced by clients arriving to mental health care through non-private systems.

Future research should aim to recruit a larger and more diverse group of clinicians to better identify the typical approach to how clinicians discuss physical activity with their youth clients. Accessing the perspectives of a greater number of clinicians working outside of private practice settings could also lead to more diverse responses. Future research should also include the direct investigation of strategies employed by clinicians to promote physical activity and whether the reality of the effectiveness of the strategies is consistent with clinician perspectives. A comparison between client and clinician perspectives on the role of physical activity in their mental well-being, and the factors impacting their participation in physical activity, could also be a fruitful avenue for future research to determine the accuracy of clinician perspectives.

## 5. Conclusions

This study contributes to our understanding of the perspectives of mental health clinicians regarding the barriers to and facilitators of physical activity for their youth clients. Many of the identified barriers and facilitators were within-person and related to specific mental health symptoms, motivation, thought patterns, screen time use, knowledge, and enjoyment of physical activity. Additional barriers and facilitators were external and included caregiver and family factors, environmental and cultural factors, and financial impacts. Clinicians were clearly considering a wide range of reasons their clients may have an easier or more difficult time engaging in physical activity, and they were considering multiple aspects of each individual client. The clinicians in our study seemed to place significant value on physical activity and were well positioned to provide support for their clients to overcome barriers and capitalize on facilitators to enhance engagement in physical activity.

## Figures and Tables

**Table 1:** Barriers and facilitators to clients’ physical activity identified by clinicians by frequency of mention in the interviews. Each time a participant mentioned a particular theme, this was counted as one unit of frequency.

Barriers	Frequency
Intrapersonal	64
Depression and anxiety	19
Lack of motivation	15
Negative self-talk and cognitive rigidity	11
Eating disorders	5
Influence of Caregivers	29
Caregiver attitude to PA	19
Dependence on caregivers	8
Financial Limitations	16
Screen time	12
Environmental and Cultural	11
Lack of Time	9
**Facilitators**	**Frequency**
Enjoyment of PA	22
Knowledge of PA benefits	8
Caregiver participation in PA	6

## Data Availability

The data presented in this study are available on request from the corresponding author. The data are not publicly available due to privacy reasons given the interview-based nature of the data.

## Appendix A

Interview Questions

1. How do you use physical activity in your clinical practice? Provide an example.
2. Are there certain populations for whom you are most likely to recommend physical activity? Any populations where you would be less likely to recommend it?
3. A good deal of the literature on how clinicians talk about physical activity with their clients has focused on adult populations. Are there any practice issues that you feel are specific to working with children and adolescents?
4. What barriers do your clients face to being active? What do you think would help them become more physically active?
